# Draft Genome Sequence of *Chryseobacterium* Strain CBo1 Isolated from *Bactrocera oleae*

**DOI:** 10.1128/genomeA.00177-17

**Published:** 2017-05-04

**Authors:** Frances Blow, John Vontas, Alistair C. Darby

**Affiliations:** a Institute of Integrative Biology, University of Liverpool, Liverpool, Merseyside, United Kingdom; b Institute of Molecular Biology & Biotechnology, Foundation for Research & Technology Hellas, Heraklion, Crete, Greece; c Department of Crop Science, Agricultural University of Athens, Athens, Greece

## Abstract

Bacteria of the genus *Chryseobacterium* have previously been identified as mutualists of plants and insects. *Chryseobacterium* strain CBo1 was cultured from the gut of the agricultural pest *Bactrocera oleae* and its whole genome sequenced. This genomic resource will aid investigations into the transition of microbes between plant and invertebrate hosts.

## GENOME ANNOUNCEMENT

Bacteria of the genus *Chryseobacterium* (family *Flavobacteriaceae*) exploit a diverse range of habitats, including soil, water, and eukaryotic organisms (1). Chryseobacteria can promote growth in plants (2, 3) and have been isolated from *Oleae europaea* olive groves (4), which are also occupied by the agricultural pest *Bactrocera oleae*. Chryseobacteria constitute an element of the gut microbiota in a broad range of invertebrates, including mosquitoes (5, 6), moths (7), cockroaches (8), and termites (GenBank accession no. KF257250.1) (M.V. Suryavanshi, Y.S. Shouche, unpublished data) and have now been discovered in the gut of *B. oleae*.

*Chryseobacterium* strain CBo1 was cultured from the homogenate of 10 dissected guts from surface-sterilized adult *B. oleae*. Guts were homogenized in Schneider’s insect medium supplemented with 10% fetal bovine serum and spread onto brain heart infusion (BHI) agar plates. The plates were incubated at 25°C for 72 h and individual colonies subsequently streaked onto BHI plates and incubated at 25°C for 72 h. DNA was isolated from single colonies by boiling at 95°C for 5 min and used as the template for PCR of the 16S rRNA gene with the primers A16SF (5′-AGAGTTTGATCMTGGCTCAG-3′) and B16SR (5′-CCCCTACGGTTACCTTGTTACGAC-3′). Sanger sequencing was performed on the resulting fragment to identify the genus of bacterium as *Chryseobacterium*. Single colonies were inoculated into BHI broth and incubated at 25°C for 72 h, and genomic DNA was extracted using the Zymo Quick DNA universal kit (Zymo), according to the manufacturer’s instructions for biological fluids and cells. The following amendments to the protocol were employed: samples were incubated with proteinase K at 55°C for 30 min rather than 10 min and were eluted twice in a volume of 40 μl to give a total of 80 μl per sample. Library preparation was performed with the NEBNext Ultra DNA library preparation kit (New England BioLabs), according to the manufacturer’s instructions, and sequencing was performed on an Illumina MiSeq sequencer at the Centre for Genomic Research, University of Liverpool, with paired-end 250-bp reads.

The resulting 2,122,794 reads were assembled with SPAdes version 3.7.1 (9). SPAdes generated a 4.5-Mb assembly comprising 71 contigs, with an *N*_50_ of 143,840 bp and an average G+C content of 35.7%. Genes were annotated using PROKKA version 1.5.2 (10), which produced a total of 4,144 protein-coding and 76 RNA genes.

In combination with draft genome sequences from other members of the *B. oleae* gut microbiota (11, 12; F. Blow, M. Koukidou, J. Vontas, A.C. Darby, unpublished data). This draft genome sequence of *Chryseobacterium* CBo1 will allow further investigation into the interactions between insects and their microbial communities. These genomic resources will also allow us to examine the transition that microbes undergo when shifting between plant and animal hosts on a range of evolutionary timescales.

### Accession number(s).

This whole-genome shotgun project has been deposited at DDBJ/ENA/GenBank under the accession no. MAUH00000000. The version described in this paper is version MAUH01000000.
